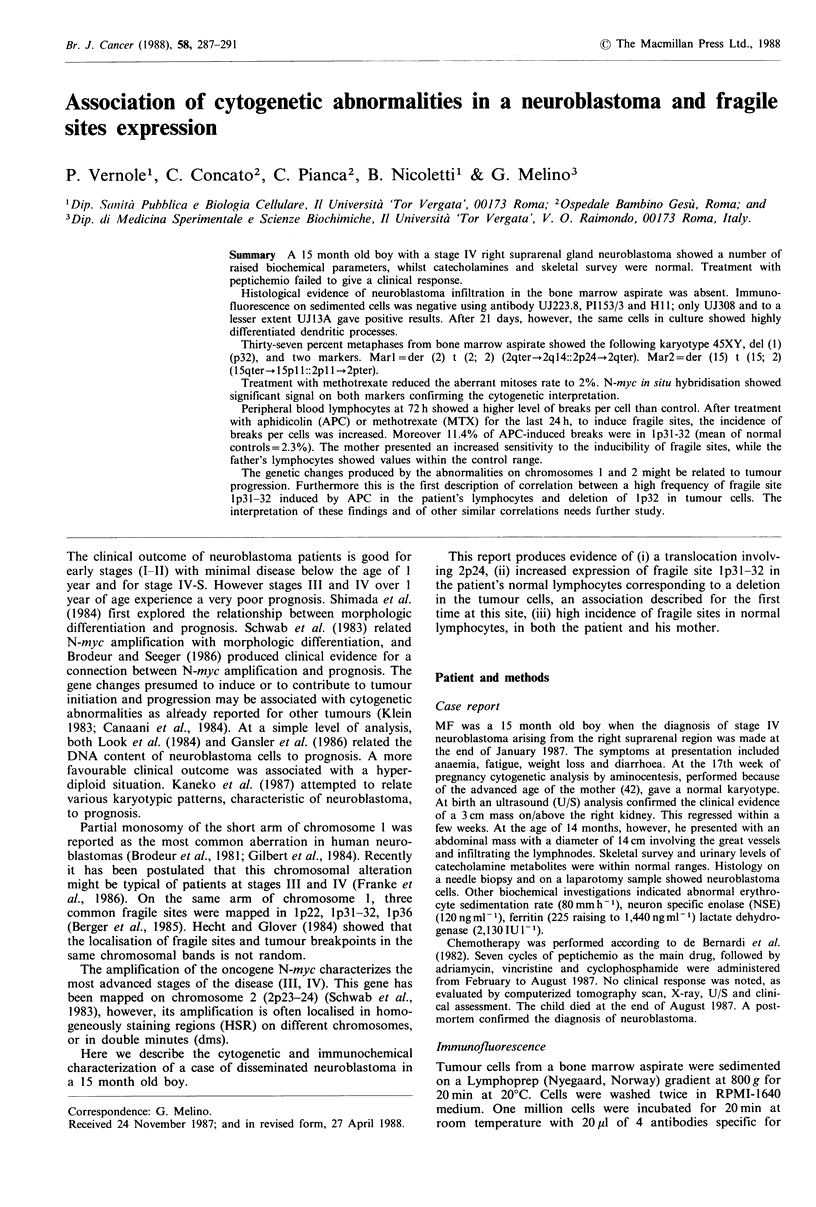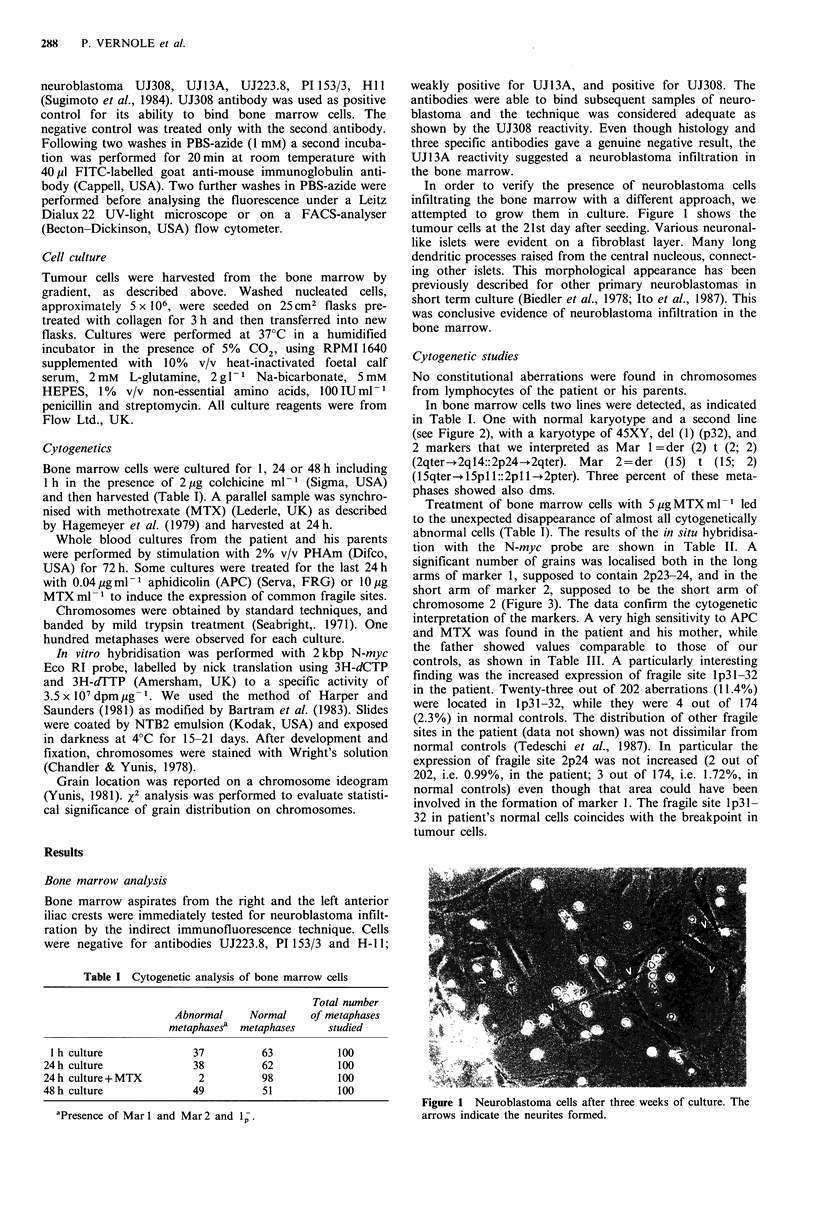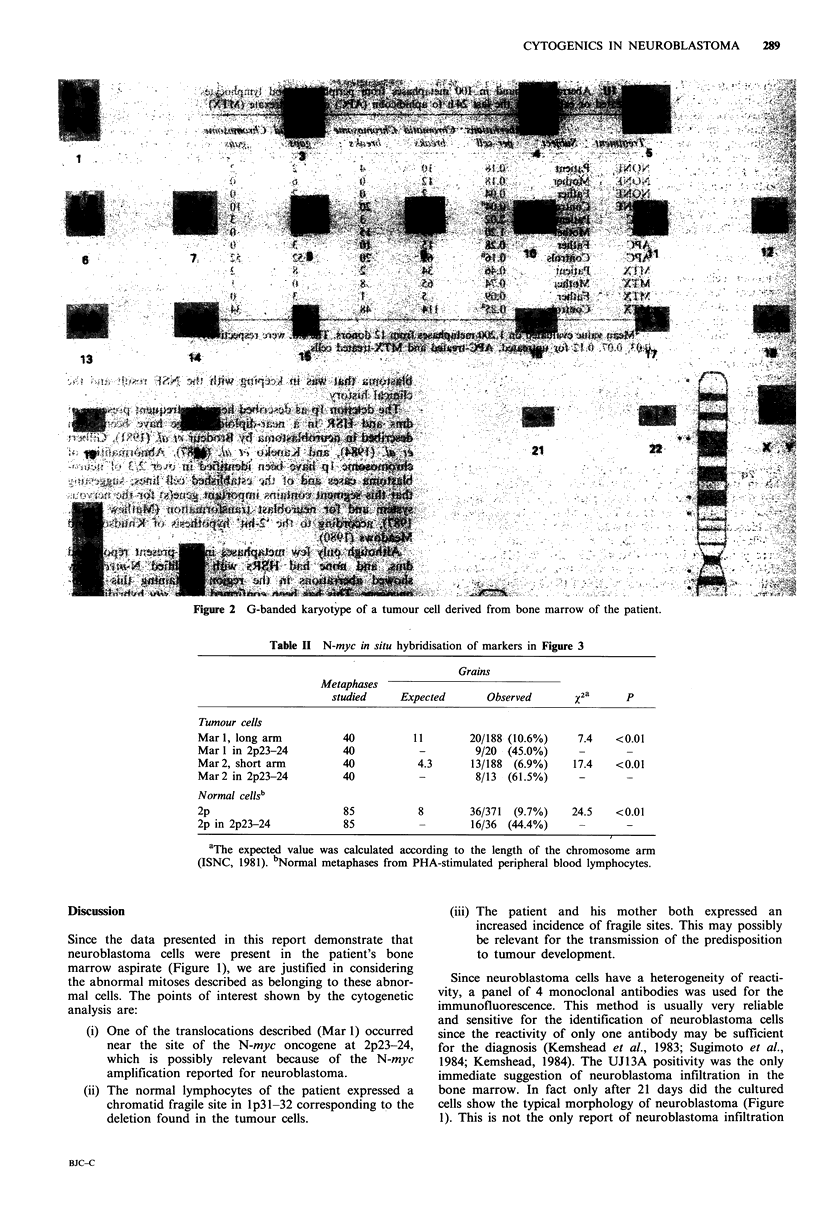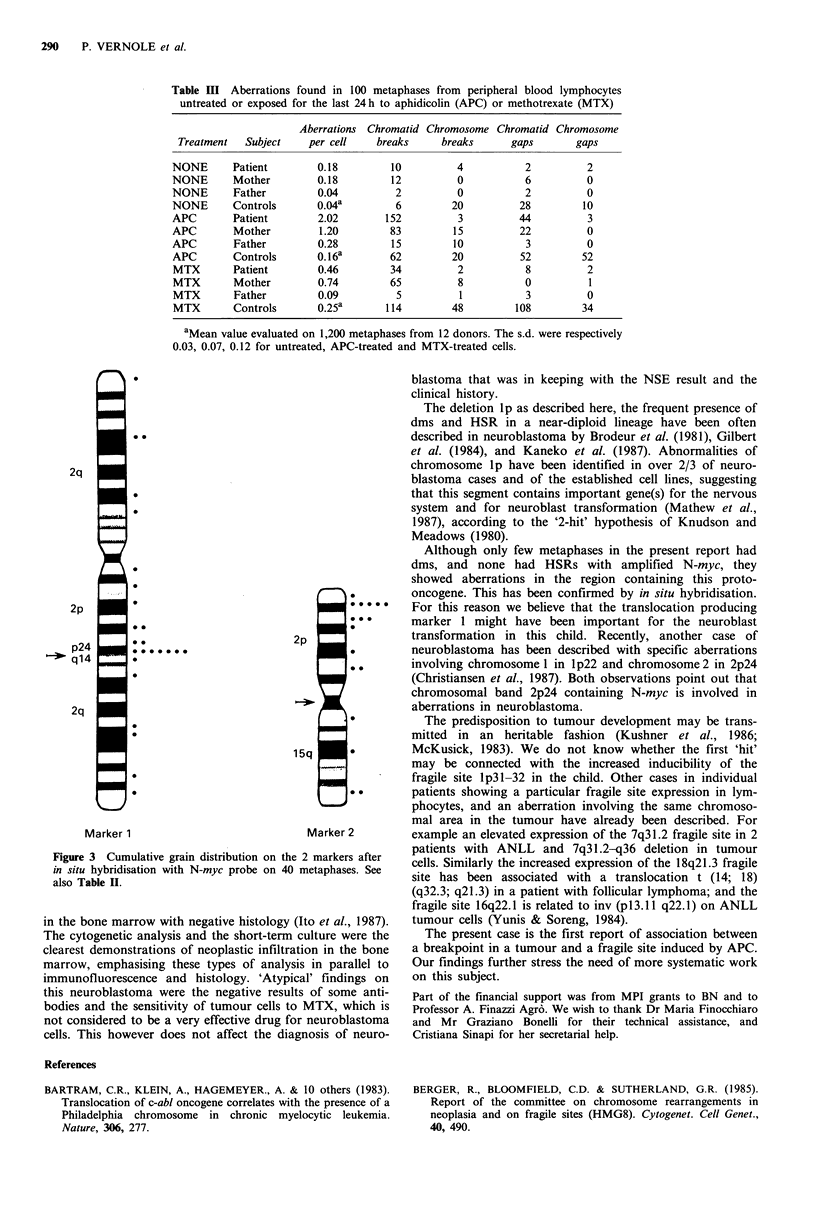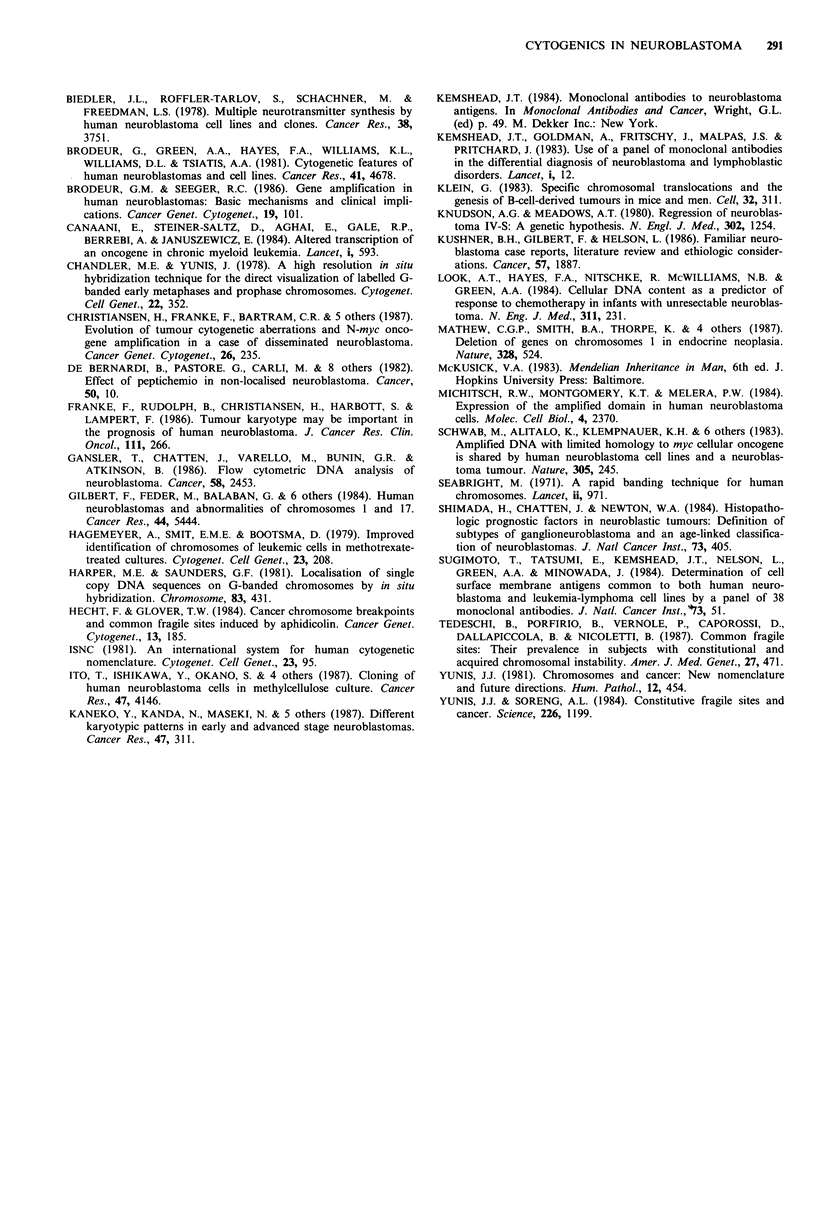# Association of cytogenetic abnormalities in a neuroblastoma and fragile sites expression.

**DOI:** 10.1038/bjc.1988.205

**Published:** 1988-09

**Authors:** P. Vernole, C. Concato, C. Pianca, B. Nicoletti, G. Melino

**Affiliations:** Dip. SanitÃ Pubblica e Biologia Cellulare, Il UniversitÃ Tor Vergata, Roma, Italy.

## Abstract

**Images:**


*Br. J. Cancer* (1988), **58**, 287–291 © The Macmillan Press Ltd., 1988

### Association of cytogenetic abnormalities in a neuroblastoma and fragile sites expression

P. Vernole[1], C. Concato[2], C. Pianca[2], B. Nicoletti[1] & G. Melino[3]

[1]*Dip. Sanità Pubblica e Biologia Cellulare, II Università 'Tor Vergata', 00173 Roma;* [2]*Ospedale Bambino Gesù, Roma; and* [3]*Dip. di Medicina Sperimentale e Scienze Biochimiche, II Università 'Tor Vergata', V. O. Raimondo, 00173 Roma, Italy.*

**Summary** A 15 month old boy with a stage IV right suprarenal gland neuroblastoma showed a number of raised biochemical parameters, whilst catecholamines and skeletal survey were normal. Treatment with peptichemio failed to give a clinical response.

Histological evidence of neuroblastoma infiltration in the bone marrow aspirate was absent. Immunofluorescence on sedimented cells was negative using antibody UJ223.8, PI153/3 and H11; only UJ308 and to a lesser extent UJ13A gave positive results. After 21 days, however, the same cells in culture showed highly differentiated dendritic processes.

Thirty-seven percent metaphases from bone marrow aspirate showed the following karyotype 45XY, del (1) (p32), and two markers. Mar1 = der (2) t (2; 2) (2qter→2q14::2p24→2qter). Mar2 = der (15) t (15; 2) (15qter→15p11::2p11→2pter).

Treatment with methotrexate reduced the aberrant mitoses rate to 2%. N-*myc in situ* hybridisation showed significant signal on both markers confirming the cytogenetic interpretation.

Peripheral blood lymphocytes at 72 h showed a higher level of breaks per cell than control. After treatment with aphidicolin (APC) or methotrexate (MTX) for the last 24 h, to induce fragile sites, the incidence of breaks per cells was increased. Moreover 11.4% of APC-induced breaks were in 1p31–32 (mean of normal controls = 2.3%). The mother presented an increased sensitivity to the inducibility of fragile sites, while the father's lymphocytes showed values within the control range.

The genetic changes produced by the abnormalities on chromosomes 1 and 2 might be related to tumour progression. Furthermore this is the first description of correlation between a high frequency of fragile site 1p31–32 induced by APC in the patient's lymphocytes and deletion of 1p32 in tumour cells. The interpretation of these findings and of other similar correlations needs further study.

The clinical outcome of neuroblastoma patients is good for early stages (I–II) with minimal disease below the age of 1 year and for stage IV-S. However stages III and IV over 1 year of age experience a very poor prognosis. Shimada *et al.* (1984) first explored the relationship between morphologic differentiation and prognosis. Schwab *et al.* (1983) related N-*myc* amplification with morphologic differentiation, and Brodeur and Seeger (1986) produced clinical evidence for a connection between N-*myc* amplification and prognosis. The gene changes presumed to induce or to contribute to tumour initiation and progression may be associated with cytogenetic abnormalities as already reported for other tumours (Klein 1983; Canaani *et al.*, 1984). At a simple level of analysis, both Look *et al.* (1984) and Gansler *et al.* (1986) related the DNA content of neuroblastoma cells to prognosis. A more favourable clinical outcome was associated with a hyperdiploid situation. Kaneko *et al.* (1987) attempted to relate various karyotypic patterns, characteristic of neuroblastoma, to prognosis.

Partial monosomy of the short arm of chromosome 1 was reported as the most common aberration in human neuroblastomas (Brodeur *et al.*, 1981; Gilbert *et al.*, 1984). Recently it has been postulated that this chromosomal alteration might be typical of patients at stages III and IV (Franke *et al.*, 1986). On the same arm of chromosome 1, three common fragile sites were mapped in 1p22, 1p31–32, 1p36 (Berger *et al.*, 1985). Hecht and Glover (1984) showed that the localisation of fragile sites and tumour breakpoints in the same chromosomal bands is not random.

The amplification of the oncogene N-*myc* characterizes the most advanced stages of the disease (III, IV). This gene has been mapped on chromosome 2 (2p23–24) (Schwab *et al.*, 1983), however, its amplification is often localised in homogeneously staining regions (HSR) on different chromosomes, or in double minutes (dms).

Here we describe the cytogenetic and immunochemical characterization of a case of disseminated neuroblastoma in a 15 month old boy.

Correspondence: G. Melino.
Received 24 November 1987; and in revised form, 27 April 1988.

This report produces evidence of (i) a translocation involving 2p24, (ii) increased expression of fragile site 1p31–32 in the patient's normal lymphocytes corresponding to a deletion in the tumour cells, an association described for the first time at this site, (iii) high incidence of fragile sites in normal lymphocytes, in both the patient and his mother.

### Patient and methods

### *Case report*

MF was a 15 month old boy when the diagnosis of stage IV neuroblastoma arising from the right suprarenal region was made at the end of January 1987. The symptoms at presentation included anaemia, fatigue, weight loss and diarrhoea. At the 17th week of pregnancy cytogenetic analysis by aminocentesis, performed because of the advanced age of the mother (42), gave a normal karyotype. At birth an ultrasound (U/S) analysis confirmed the clinical evidence of a 3 cm mass on/above the right kidney. This regressed within a few weeks. At the age of 14 months, however, he presented with an abdominal mass with a diameter of 14 cm involving the great vessels and infiltrating the lymphnodes. Skeletal survey and urinary levels of catecholamine metabolites were within normal ranges. Histology on a needle biopsy and on a laparotomy sample showed neuroblastoma cells. Other biochemical investigations indicated abnormal erythrocyte sedimentation rate (80 mm $h^{-1}$), neuron specific enolase (NSE) (120 ng $ml^{-1}$), ferritin (225 raising to 1,440 ng $ml^{-1}$) lactate dehydrogenase (2,130 IU $l^{-1}$).

Chemotherapy was performed according to de Bernardi *et al.* (1982). Seven cycles of peptichemio as the main drug, followed by adriamycin, vincristine and cyclophosphamide were administered from February to August 1987. No clinical response was noted, as evaluated by computerized tomography scan, X-ray, U/S and clinical assessment. The child died at the end of August 1987. A postmortem confirmed the diagnosis of neuroblastoma.

### *Immunofluorescence*

Tumour cells from a bone marrow aspirate were sedimented on a Lymphoprep (Nyegaard, Norway) gradient at 800 *g* for 20 min at 20°C. Cells were washed twice in RPMI-1640 medium. One million cells were incubated for 20 min at room temperature with 20 μl of 4 antibodies specific for neuroblastoma UJ308, UJ13A, UJ223.8, PI 153/3, H11 (Sugimoto *et al.*, 1984). UJ308 antibody was used as positive control for its ability to bind bone marrow cells. The negative control was treated only with the second antibody. Following two washes in PBS-azide (1 mM) a second incubation was performed for 20 min at room temperature with 40 $\mu$l FITC-labelled goat anti-mouse immunoglobulin antibody (Cappell, USA). Two further washes in PBS-azide were performed before analysing the fluorescence under a Leitz Dialux 22 UV-light microscope or on a FACS-analyser (Becton–Dickinson, USA) flow cytometer.

*Cell culture*

Tumour cells were harvested from the bone marrow by gradient, as described above. Washed nucleated cells, approximately $5 \times 10^6$, were seeded on 25 $cm^2$ flasks pretreated with collagen for 3 h and then transferred into new flasks. Cultures were performed at 37°C in a humidified incubator in the presence of 5% $CO_2$, using RPMI 1640 supplemented with 10% v/v heat-inactivated foetal calf serum, 2 mM L-glutamine, 2 $g l^{-1}$ Na-bicarbonate, 5 mM HEPES, 1% v/v non-essential amino acids, 100 IU $ml^{-1}$ penicillin and streptomycin. All culture reagents were from Flow Ltd., UK.

*Cytogenetics*

Bone marrow cells were cultured for 1, 24 or 48 h including 1 h in the presence of 2 $\mu$g colchicine $ml^{-1}$ (Sigma, USA) and then harvested (Table I). A parallel sample was synchronised with methotrexate (MTX) (Lederle, UK) as described by Hagemeyer *et al.* (1979) and harvested at 24 h.

Whole blood cultures from the patient and his parents were performed by stimulation with 2% v/v PHAm (Difco, USA) for 72 h. Some cultures were treated for the last 24 h with 0.04 $\mu g\,ml^{-1}$ aphidicolin (APC) (Serva, FRG) or 10 $\mu$g MTX $ml^{-1}$ to induce the expression of common fragile sites.

Chromosomes were obtained by standard techniques, and banded by mild trypsin treatment (Seabright,. 1971). One hundred metaphases were observed for each culture.

*In vitro* hybridisation was performed with 2 kbp N-*myc* Eco RI probe, labelled by nick translation using 3H-*d*CTP and 3H-*d*TTP (Amersham, UK) to a specific activity of $3.5 \times 10^7$ dpm $\mu g^{-1}$. We used the method of Harper and Saunders (1981) as modified by Bartram *et al.* (1983). Slides were coated by NTB2 emulsion (Kodak, USA) and exposed in darkness at 4°C for 15–21 days. After development and fixation, chromosomes were stained with Wright's solution (Chandler & Yunis, 1978).

Grain location was reported on a chromosome ideogram (Yunis, 1981). $\chi^2$ analysis was performed to evaluate statistical significance of grain distribution on chromosomes.

### Results

*Bone marrow analysis*

Bone marrow aspirates from the right and the left anterior iliac crests were immediately tested for neuroblastoma infiltration by the indirect immunofluorescence technique. Cells were negative for antibodies UJ223.8, PI 153/3 and H-11; weakly positive for UJ13A, and positive for UJ308. The antibodies were able to bind subsequent samples of neuroblastoma and the technique was considered adequate as shown by the UJ308 reactivity. Even though histology and three specific antibodies gave a genuine negative result, the UJ13A reactivity suggested a neuroblastoma infiltration in the bone marrow.

In order to verify the presence of neuroblastoma cells infiltrating the bone marrow with a different approach, we attempted to grow them in culture. Figure 1 shows the tumour cells at the 21st day after seeding. Various neuronal-like islets were evident on a fibroblast layer. Many long dendritic processes raised from the central nucleous, connecting other islets. This morphological appearance has been previously described for other primary neuroblastomas in short term culture (Biedler *et al.*, 1978; Ito *et al.*, 1987). This was conclusive evidence of neuroblastoma infiltration in the bone marrow.

**Table I** Cytogenetic analysis of bone marrow cells

| | *Abnormal metaphases*[a] | *Normal metaphases* | *Total number of metaphases studied* |
|---|---|---|---|
| 1 h culture | 37 | 63 | 100 |
| 24 h culture | 38 | 62 | 100 |
| 24 h culture + MTX | 2 | 98 | 100 |
| 48 h culture | 49 | 51 | 100 |

[a]Presence of Mar 1 and Mar 2 and $1_p^-$.

*Cytogenetic studies*

No constitutional aberrations were found in chromosomes from lymphocytes of the patient or his parents.

In bone marrow cells two lines were detected, as indicated in Table I. One with normal karyotype and a second line (see Figure 2), with a karyotype of 45XY, del (1) (p32), and 2 markers that we interpreted as Mar 1 = der (2) t (2; 2) (2qter→2q14::2p24→2qter). Mar 2 = der (15) t (15; 2) (15qter→15p11::2p11→2pter). Three percent of these metaphases showed also dms.

Treatment of bone marrow cells with 5 $\mu$g MTX $ml^{-1}$ led to the unexpected disappearance of almost all cytogenetically abnormal cells (Table I). The results of the *in situ* hybridisation with the N-*myc* probe are shown in Table II. A significant number of grains was localised both in the long arms of marker 1, supposed to contain 2p23–24, and in the short arm of marker 2, supposed to be the short arm of chromosome 2 (Figure 3). The data confirm the cytogenetic interpretation of the markers. A very high sensitivity to APC and MTX was found in the patient and his mother, while the father showed values comparable to those of our controls, as shown in Table III. A particularly interesting finding was the increased expression of fragile site 1p31–32 in the patient. Twenty-three out of 202 aberrations (11.4%) were located in 1p31–32, while they were 4 out of 174 (2.3%) in normal controls. The distribution of other fragile sites in the patient (data not shown) was not dissimilar from normal controls (Tedeschi *et al.*, 1987). In particular the expression of fragile site 2p24 was not increased (2 out of 202, i.e. 0.99%, in the patient; 3 out of 174, i.e. 1.72%, in normal controls) even though that area could have been involved in the formation of marker 1. The fragile site 1p31–32 in patient's normal cells coincides with the breakpoint in tumour cells.

**Figure 1** Neuroblastoma cells after three weeks of culture. The arrows indicate the neurites formed.

**Figure 2** G-banded karyotype of a tumour cell derived from bone marrow of the patient.

**Table II** N-*myc in situ* hybridisation of markers in **Figure 3**

| | *Metaphases studied* | *Grains* | | $\chi^{2a}$ | *P* |
|---|---|---|---|---|---|
| | | *Expected* | *Observed* | | |
| *Tumour cells* | | | | | |
| Mar 1, long arm | 40 | 11 | 20/188 (10.6%) | 7.4 | <0.01 |
| Mar 1 in 2p23–24 | 40 | – | 9/20 (45.0%) | – | – |
| Mar 2, short arm | 40 | 4.3 | 13/188 (6.9%) | 17.4 | <0.01 |
| Mar 2 in 2p23–24 | 40 | – | 8/13 (61.5%) | – | – |
| *Normal cells*[b] | | | | | |
| 2p | 85 | 8 | 36/371 (9.7%) | 24.5 | <0.01 |
| 2p in 2p23–24 | 85 | – | 16/36 (44.4%) | – | – |

[a]The expected value was calculated according to the length of the chromosome arm (ISNC, 1981). [b]Normal metaphases from PHA-stimulated peripheral blood lymphocytes.

### Discussion

Since the data presented in this report demonstrate that neuroblastoma cells were present in the patient's bone marrow aspirate (Figure 1), we are justified in considering the abnormal mitoses described as belonging to these abnormal cells. The points of interest shown by the cytogenetic analysis are:

(i) One of the translocations described (Mar 1) occurred near the site of the N-*myc* oncogene at 2p23–24, which is possibly relevant because of the N-*myc* amplification reported for neuroblastoma.

(ii) The normal lymphocytes of the patient expressed a chromatid fragile site in 1p31–32 corresponding to the deletion found in the tumour cells.

(iii) The patient and his mother both expressed an increased incidence of fragile sites. This may possibly be relevant for the transmission of the predisposition to tumour development.

Since neuroblastoma cells have a heterogeneity of reactivity, a panel of 4 monoclonal antibodies was used for the immunofluorescence. This method is usually very reliable and sensitive for the identification of neuroblastoma cells since the reactivity of only one antibody may be sufficient for the diagnosis (Kemshead *et al.*, 1983; Sugimoto *et al.*, 1984; Kemshead, 1984). The UJ13A positivity was the only immediate suggestion of neuroblastoma infiltration in the bone marrow. In fact only after 21 days did the cultured cells show the typical morphology of neuroblastoma (Figure 1). This is not the only report of neuroblastoma infiltration

**Table III** Aberrations found in 100 metaphases from peripheral blood lymphocytes untreated or exposed for the last 24 h to aphidicolin (APC) or methotrexate (MTX)

| *Treatment* | *Subject* | *Aberrations per cell* | *Chromatid breaks* | *Chromosome breaks* | *Chromatid gaps* | *Chromosome gaps* |
|---|---|---|---|---|---|---|
| NONE | Patient | 0.18 | 10 | 4 | 2 | 2 |
| NONE | Mother | 0.18 | 12 | 0 | 6 | 0 |
| NONE | Father | 0.04 | 2 | 0 | 2 | 0 |
| NONE | Controls | 0.04[a] | 6 | 20 | 28 | 10 |
| APC | Patient | 2.02 | 152 | 3 | 44 | 3 |
| APC | Mother | 1.20 | 83 | 15 | 22 | 0 |
| APC | Father | 0.28 | 15 | 10 | 3 | 0 |
| APC | Controls | 0.16[a] | 62 | 20 | 52 | 52 |
| MTX | Patient | 0.46 | 34 | 2 | 8 | 2 |
| MTX | Mother | 0.74 | 65 | 8 | 0 | 1 |
| MTX | Father | 0.09 | 5 | 1 | 3 | 0 |
| MTX | Controls | 0.25[a] | 114 | 48 | 108 | 34 |

[a]Mean value evaluated on 1,200 metaphases from 12 donors. The s.d. were respectively 0.03, 0.07, 0.12 for untreated, APC-treated and MTX-treated cells.

**Figure 3** Cumulative grain distribution on the 2 markers after *in situ* hybridisation with N-*myc* probe on 40 metaphases. See also **Table II**.

in the bone marrow with negative histology (Ito *et al.*, 1987). The cytogenetic analysis and the short-term culture were the clearest demonstrations of neoplastic infiltration in the bone marrow, emphasising these types of analysis in parallel to immunofluorescence and histology. 'Atypical' findings on this neuroblastoma were the negative results of some antibodies and the sensitivity of tumour cells to MTX, which is not considered to be a very effective drug for neuroblastoma cells. This however does not affect the diagnosis of neuroblastoma that was in keeping with the NSE result and the clinical history.

The deletion 1p as described here, the frequent presence of dms and HSR in a near-diploid lineage have been often described in neuroblastoma by Brodeur *et al.* (1981), Gilbert *et al.* (1984), and Kaneko *et al.* (1987). Abnormalities of chromosome 1p have been identified in over 2/3 of neuroblastoma cases and of the established cell lines, suggesting that this segment contains important gene(s) for the nervous system and for neuroblast transformation (Mathew *et al.*, 1987), according to the '2-hit' hypothesis of Knudson and Meadows (1980).

Although only few metaphases in the present report had dms, and none had HSRs with amplified N-*myc*, they showed aberrations in the region containing this proto-oncogene. This has been confirmed by *in situ* hybridisation. For this reason we believe that the translocation producing marker 1 might have been important for the neuroblast transformation in this child. Recently, another case of neuroblastoma has been described with specific aberrations involving chromosome 1 in 1p22 and chromosome 2 in 2p24 (Christiansen *et al.*, 1987). Both observations point out that chromosomal band 2p24 containing N-*myc* is involved in aberrations in neuroblastoma.

The predisposition to tumour development may be transmitted in an heritable fashion (Kushner *et al.*, 1986; McKusick, 1983). We do not know whether the first 'hit' may be connected with the increased inducibility of the fragile site 1p31–32 in the child. Other cases in individual patients showing a particular fragile site expression in lymphocytes, and an aberration involving the same chromosomal area in the tumour have already been described. For example an elevated expression of the 7q31.2 fragile site in 2 patients with ANLL and 7q31.2–q36 deletion in tumour cells. Similarly the increased expression of the 18q21.3 fragile site has been associated with a translocation t (14; 18) (q32.3; q21.3) in a patient with follicular lymphoma; and the fragile site 16q22.1 is related to inv (p13.11 q22.1) on ANLL tumour cells (Yunis & Soreng, 1984).

The present case is the first report of association between a breakpoint in a tumour and a fragile site induced by APC. Our findings further stress the need of more systematic work on this subject.

Part of the financial support was from MPI grants to BN and to Professor A. Finazzi Agrò. We wish to thank Dr Maria Finocchiaro and Mr Graziano Bonelli for their technical assistance, and Cristiana Sinapi for her secretarial help.

### References

BARTRAM, C.R., KLEIN, A., HAGEMEYER., A. & 10 others (1983). Translocation of c-*abl* oncogene correlates with the presence of a Philadelphia chromosome in chronic myelocytic leukemia. *Nature*, **306**, 277.

BERGER, R., BLOOMFIELD, C.D. & SUTHERLAND, G.R. (1985). Report of the committee on chromosome rearrangements in neoplasia and on fragile sites (HMG8). *Cytogenet. Cell Genet.*, **40**, 490.

BIEDLER, J.L., ROFFLER-TARLOV, S., SCHACHNER, M. & FREEDMAN, L.S. (1978). Multiple neurotransmitter synthesis by human neuroblastoma cell lines and clones. *Cancer Res.*, **38**, 3751.

BRODEUR, G., GREEN, A.A., HAYES, F.A., WILLIAMS, K.L., WILLIAMS, D.L. & TSIATIS, A.A. (1981). Cytogenetic features of human neuroblastomas and cell lines. *Cancer Res.*, **41**, 4678.

BRODEUR, G.M. & SEEGER, R.C. (1986). Gene amplification in human neuroblastomas: Basic mechanisms and clinical implications. *Cancer Genet. Cytogenet.*, **19**, 101.

CANAANI, E., STEINER-SALTZ, D., AGHAI, E., GALE, R.P., BERREBI, A. & JANUSZEWICZ, E. (1984). Altered transcription of an oncogene in chronic myeloid leukemia. *Lancet*, **i**, 593.

CHANDLER, M.E. & YUNIS, J. (1978). A high resolution *in situ* hybridization technique for the direct visualization of labelled G-banded early metaphases and prophase chromosomes. *Cytogenet. Cell Genet.*, **22**, 352.

CHRISTIANSEN, H., FRANKE, F., BARTRAM, C.R. & 5 others (1987). Evolution of tumour cytogenetic aberrations and N-*myc* oncogene amplification in a case of disseminated neuroblastoma. *Cancer Genet. Cytogenet.*, **26**, 235.

DE BERNARDI, B., PASTORE. G., CARLI, M. & 8 others (1982). Effect of peptichemio in non-localised neuroblastoma. *Cancer*, **50**, 10.

FRANKE, F., RUDOLPH, B., CHRISTIANSEN, H., HARBOTT, S. & LAMPERT, F. (1986). Tumour karyotype may be important in the prognosis of human neuroblastoma. *J. Cancer Res. Clin. Oncol.*, **111**, 266.

GANSLER, T., CHATTEN, J., VARELLO, M., BUNIN, G.R. & ATKINSON, B. (1986). Flow cytometric DNA analysis of neuroblastoma. *Cancer*, **58**, 2453.

GILBERT, F., FEDER, M., BALABAN, G. & 6 others (1984). Human neuroblastomas and abnormalities of chromosomes 1 and 17. *Cancer Res.*, **44**, 5444.

HAGEMEYER, A., SMIT, E.M.E. & BOOTSMA, D. (1979). Improved identification of chromosomes of leukemic cells in methotrexate-treated cultures. *Cytogenet. Cell Genet.*, **23**, 208.

HARPER, M.E. & SAUNDERS, G.F. (1981). Localisation of single copy DNA sequences on G-banded chromosomes by *in situ* hybridization. *Chromosome*, **83**, 431.

HECHT, F. & GLOVER, T.W. (1984). Cancer chromosome breakpoints and common fragile sites induced by aphidicolin. *Cancer Genet. Cytogenet.*, **13**, 185.

ISNC (1981). An international system for human cytogenetic nomenclature. *Cytogenet. Cell Genet.*, **23**, 95.

ITO, T., ISHIKAWA, Y., OKANO, S. & 4 others (1987). Cloning of human neuroblastoma cells in methylcellulose culture. *Cancer Res.*, **47**, 4146.

KANEKO, Y., KANDA, N., MASEKI, N. & 5 others (1987). Different karyotypic patterns in early and advanced stage neuroblastomas. *Cancer Res.*, **47**, 311.

KEMSHEAD, J.T. (1984). Monoclonal antibodies to neuroblastoma antigens. In *Monoclonal Antibodies and Cancer*, Wright, G.L. (ed) p. 49. M. Dekker Inc.: New York.

KEMSHEAD, J.T., GOLDMAN, A., FRITSCHY, J., MALPAS, J.S. & PRITCHARD, J. (1983). Use of a panel of monoclonal antibodies in the differential diagnosis of neuroblastoma and lymphoblastic disorders. *Lancet*, **i**, 12.

KLEIN, G. (1983). Specific chromosomal translocations and the genesis of B-cell-derived tumours in mice and men. *Cell*, **32**, 311.

KNUDSON, A.G. & MEADOWS, A.T. (1980). Regression of neuroblastoma IV-S: A genetic hypothesis. *N. Engl. J. Med.*, **302**, 1254.

KUSHNER, B.H., GILBERT, F. & HELSON, L. (1986). Familiar neuroblastoma case reports, literature review and ethiologic considerations. *Cancer*, **57**, 1887.

LOOK, A.T., HAYES, F.A., NITSCHKE, R. McWILLIAMS, N.B. & GREEN, A.A. (1984). Cellular DNA content as a predictor of response to chemotherapy in infants with unresectable neuroblastoma. *N. Eng. J. Med.*, **311**, 231.

MATHEW, C.G.P., SMITH, B.A., THORPE, K. & 4 others (1987). Deletion of genes on chromosomes 1 in endocrine neoplasia. *Nature*, **328**, 524.

McKUSICK, V.A. (1983). *Mendelian Inheritance in Man*, 6th ed. J. Hopkins University Press: Baltimore.

MICHITSCH, R.W., MONTGOMERY, K.T. & MELERA, P.W. (1984). Expression of the amplified domain in human neuroblastoma cells. *Molec. Cell Biol.*, **4**, 2370.

SCHWAB, M., ALITALO, K., KLEMPNAUER, K.H. & 6 others (1983). Amplified DNA with limited homology to *myc* cellular oncogene is shared by human neuroblastoma cell lines and a neuroblastoma tumour. *Nature*, **305**, 245.

SEABRIGHT, M. (1971). A rapid banding technique for human chromosomes. *Lancet*, **ii**, 971.

SHIMADA, H., CHATTEN, J. & NEWTON, W.A. (1984). Histopathologic prognostic factors in neuroblastic tumours: Definition of subtypes of ganglioneuroblastoma and an age-linked classification of neuroblastomas. *J. Natl Cancer Inst.*, **73**, 405.

SUGIMOTO, T., TATSUMI, E., KEMSHEAD, J.T., NELSON, L., GREEN, A.A. & MINOWADA, J. (1984). Determination of cell surface membrane antigens common to both human neuroblastoma and leukemia-lymphoma cell lines by a panel of 38 monoclonal antibodies. *J. Natl. Cancer Inst.*, **73**, 51.

TEDESCHI, B., PORFIRIO, B., VERNOLE, P., CAPOROSSI, D., DALLAPICCOLA, B. & NICOLETTI, B. (1987). Common fragile sites: Their prevalence in subjects with constitutional and acquired chromosomal instability. *Amer. J. Med. Genet.*, **27**, 471.

YUNIS, J.J. (1981). Chromosomes and cancer: New nomenclature and future directions. *Hum. Pathol.*, **12**, 454.

YUNIS, J.J. & SORENG, A.L. (1984). Constitutive fragile sites and cancer. *Science*, **226**, 1199.